# Contamination of Groundwater Systems in the US and Canada by Enteric Pathogens, 1990–2013: A Review and Pooled-Analysis

**DOI:** 10.1371/journal.pone.0093301

**Published:** 2014-05-07

**Authors:** Paul Dylan Hynds, M. Kate Thomas, Katarina Dorothy Milena Pintar

**Affiliations:** Centre for Food-borne, Environmental and Zoonotic Infectious Diseases, Public Health Agency of Canada, Ottawa, Ontario, Canada; The Australian National University, Australia

## Abstract

**Background:**

Up to 150 million North Americans currently use a groundwater system as their principal drinking water source. These systems are a potential source of exposure to enteric pathogens, contributing to the burden of waterborne disease. Waterborne disease outbreaks have been associated with US and Canadian groundwater systems over the past two decades. However, to date, this literature has not been reviewed in a comprehensive manner.

**Methods and Principal Findings:**

A combined review and pooled-analysis approach was used to investigate groundwater contamination in Canada and the US from 1990 to 2013; fifty-five studies met eligibility criteria. Four study types were identified. It was found that study location affects study design, sample rate and studied pathogen category. Approximately 15% (316/2210) of samples from Canadian and US groundwater sources were positive for enteric pathogens, with no difference observed based on system type. Knowledge gaps exist, particularly in exposure assessment for attributing disease to groundwater supplies. Furthermore, there is a lack of consistency in risk factor reporting (local hydrogeology, well type, well use, etc). The widespread use of fecal indicator organisms in reported studies does not inform the assessment of human health risks associated with groundwater supplies.

**Conclusions:**

This review illustrates how groundwater study design and location are critical for subsequent data interpretation and use. Knowledge gaps exist related to data on bacterial, viral and protozoan pathogen prevalence in Canadian and US groundwater systems, as well as a need for standardized approaches for reporting study design and results. Fecal indicators are examined as a surrogate for health risk assessments; caution is advised in their widespread use. Study findings may be useful during suspected waterborne outbreaks linked with a groundwater supply to identify the likely etiological agent and potential transport pathway.

## Introduction

The diversity of North America’s hydrology, and by extension, hydrogeology, is a reflection of its bio-physical diversity including extremely variable physiography, climate, bedrock geology and quaternary geology. Accordingly, many regional hydrological issues exist related to microbial groundwater contamination [1].

Canada is comprised of nine hydrogeological regions, ranging from extensive areas of permafrost in the north to Cordillera, the Western Canadian sedimentary basin and the St Lawrence platform in the south [2]. Approximately 8.9 million Canadians (30.3% of the population) currently rely on groundwater for domestic use; with an estimated 3.8 million Canadians served by private supplies [3]–[6]. Hydrogeological units within the United States (US) are similarly varied with approximately 69.5% of groundwater in the US derived from unconsolidated aquifers (alluvial sand and gravel, coastal plain, glacial outwash and fluvial/eolian) [7]. Approximately 44% of the U.S. population depends on groundwater for its drinking water supply [8]. Private wells constitute the largest proportion of water wells in the United States – more than 13.2 million households have their own well, supplying 45 million people [8]. Typically, groundwater supplies in Canada and the United States are categorized into one of three supply types; large-scale, disinfected municipal systems supplying cities and municipalities with >1000 residents, community systems of varying scale with or without disinfection and typically supplying <1000 people, and largely untreated private domestic wells, often serving single households.

Groundwater contamination occurs as a direct result of human or animal waste ingress to groundwater resources (aquifers or wells). Enteric pathogens are prevalent in both developing and developed countries, including the United States and Canada [9]–[10], where septic tanks [11]–[13], sewage and municipal wastewater treatment [14]–[16], wildlife [17], grazing animals [18] and other agricultural activities [19]–[22] can be sources of groundwater contamination. Natural attenuation occurs by overlying subsoil strata; however, age deterioration, poor wellhead hygiene or design defects provide localized pollution pathways [13], [23]. Bedrock fracturing and faulting, unconsolidated bedrock materials and thin or absent overlying subsoil layers may also provide pathways for contamination transport. Where subsurface contamination does occur, adverse human health effects may result, in part due to inadequate treatment, particularly with private supplies [24], [25].

Waterborne illness is a public health issue in North America and around the world. Sensitive sub-populations including the young, the elderly, pregnant women, and the immune-compromised are particularly susceptible to enteric infections [26]. An estimated 45% of all waterborne outbreaks in Canada involve non-municipal systems, largely in rural or remote areas [3], [4], [27]. While private supplies serve about 13% of Canada's population, >20% of Canada's reported waterborne disease outbreaks were associated with private water supplies between 1974 and 1996 [28]. Consumption from private wells has been cited as a potentially significant risk factor for contracting campylobacteriosis and other enteric diseases in Canada [29]–[31]. A recent study from New Zealand predicts that consumers sourcing their drinking water supply from shallow groundwater in an intensive dairy region (Waikakahi catchment, Canterbury) had an estimated 60–75% risk of *Campylobacter* infection per irrigation season [22]. Consumption of contaminated groundwater is estimated to account for approximately 6.5 million illnesses per year in the US (34% of all waterborne infections) [32]. A recent review by Wallender *et al*. [16] found that 30.3% of 818 drinking water outbreaks reported to the United States CDC Waterborne Disease and Outbreak Surveillance Systems during 1971 to 2008 were attributable to untreated groundwater sources. Exposure to shallow groundwater sources contaminated with animal faeces has been identified as a risk factor for *Escherichia coli* O157:H7 infection in the US [33].

In their summation of a 2005 workshop entitled ‘*Estimating Waterborne Disease Risks in the United States*’ Craun et al. [34] state that significant data and method-based uncertainties remain in the improved estimation of enteric diseases attributed to drinking water, particularly with respect to risks associated with wells and groundwater. There is currently a paucity of integrated data pertaining to the presence and source of enteric pathogens in North American groundwater systems. Accordingly, the purpose of this work was to identify and review available literature relating to contamination of Canadian and US groundwater sources by enteric pathogens between 1990 and 2013. The overarching objective of this review was a comprehensive collation of the current state of knowledge relating to the presence of enteric pathogens in Canadian and US groundwater and an examination of related patterns (e.g. use of FIOs, enteric pathogen detection, study location, source types, etc.). To date, similar work has not been undertaken; it is considered that the results could be used by stakeholders to prioritize research and fill existing data gaps, thus supporting the effective management of groundwater supplies in Canada and the US.

## Methods

### 2.1 Literature Identification and Screening

The research question that guided the review was:

*What has been the prevalence of contamination by enteric pathogens and fecal indicator organisms in Canadian and US groundwater systems during the period 1990-present?*

**Figure 1:**
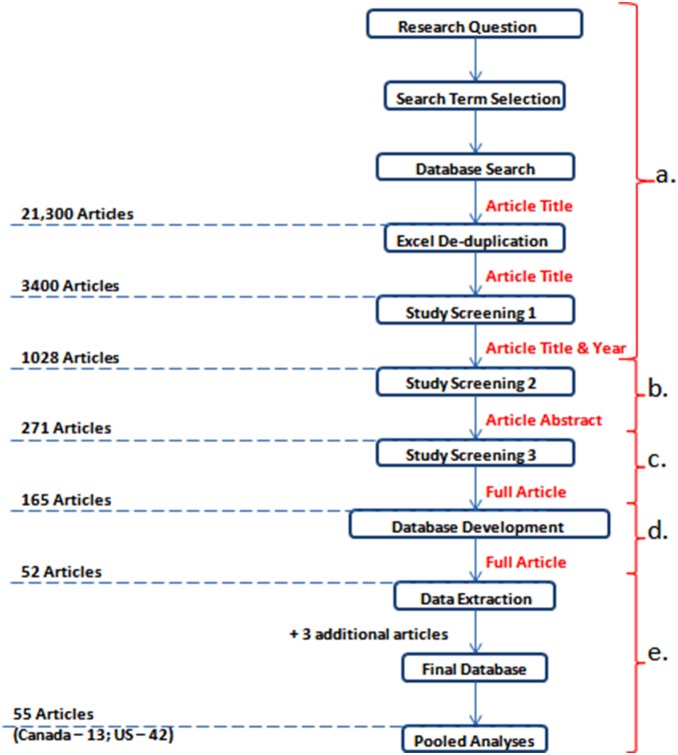
Review protocol employed throughout the current study, including results of literature identification, literature scans and data extraction processes.

A review protocol was developed in order to capture existing, published references to contamination events in Canadian and US groundwater systems (Figure 1). The review protocol was adapted from previous studies [35]–[37]. Literature for inclusion was identified from four bibliographic databases (PubMed, MedLine Plus, Science Direct, and Scopus) with key search terms (Table 1). Overall, 21,300 article titles that included ≥2 key search terms were located within bibliographic databases; article titles were imported to an Excel spreadsheet and de-duplicated using a replacement macro, resulting in 3400 article titles (Figure 1a). All literature scans were undertaken using Boolean positional operators (“AND”, “OR”, “SAME”, “WITH”, “ADJ”) to focus literature searches. Study screening 1 (citation title) was subsequently undertaken; all articles published prior to 1990, relating to groundwater chemistry, non-enteric pathogens and/or groundwater systems outside of Canada and the US were excluded. This resulted in the inclusion of 1028 non-duplicate articles (Figure 1b).

**Table 1:** Review primary terms, term descriptions and search terms used for literature identification, study screening and final database development.

Primary Term	Term Description	Search Terms
**Population**		
**Groundwater**	Environment/Pathway of interest	Groundwater; Aquifer; Well; Waterborne
**Jurisdiction**	Geographical area of interest	USA/Canada/North America
**Outcome**		
**Contamination**	Outcome of interest	Contamination; Pollution; Runoff; Faecal Ingress, Presence
**Agent**		
**Pathogen**	Organisms of interest	Pathogen(ic); Enteric; Virus; Protozoa; Bacteria;Microbial, Indicator, *Giardia; Cryptosporidium;* *Shigella;* STEC; VTEC; *E. coli*; Adenovirus;Enterovirus *Campylobacter;* Norovirus; Rotavirus

Study screening 2 (citation abstract) was based upon explicit inclusion/exclusion criteria (Table 2). No study types were excluded, however, at this stage all non-English articles were excluded, as were articles which examined surface, recreational or coastal waterbodies. Articles reporting groundwater contamination by non-enteric pathogens were also excluded during this screening phase. This screening phase was undertaken by one researcher, with 270 article included for further consideration at this stage (Figure 1c).

**Table 2:** Inclusion and exclusion criteria for literature screening.

Inclusion Criteria	Exclusion Criteria
Study Type: All	Study Type: None
Language: English	Language: Non English
Population: GroundwaterSystems (Municipal,Private, Community,Non-community, Mixed)	Population: Surface waterbodies/watersheds, coastal/marinewater bodies, recreational water
Events/Outcomes: Confirmed contamination ofgroundwater systems by enteric pathogens inNorth America; Confirmed presence offecal indicator organisms inNorth Americangroundwater systems	Events/Outcomes: Groundwatercontamination occurring outsideNorth America,groundwater contaminationby “non-enteric” pathogens
Period: 1990 – present	Period: pre-1990

Study screening 3 was completed by two review teams who independently assessed each publication (year, title, and abstract), based upon explicit eligibility criteria (Table 2). Articles that met all initial screening criteria, as defined by agreement between reviewers were included. Disagreements (approximately 15%) were resolved by discussion among reviewers and an additional round of screening. As shown (Figure 1d), 165 articles were included in the final database. Upon data extraction, 52 of 165 included articles were found to contain extractable homogenous data, with an additional three relevant articles located via article bibliographies added at this stage (Figure 1e).

### 2.2 Data Extraction

Relevant data were extracted to a spreadsheet (MS Office Excel 2007) and verified. Three primary data fields were extracted and exported, including:

1. Citation-specific data: Title, Author(s), Publication Year, Publication Type, Study Country.
2. Groundwater contamination data: Fecal Indicator Organism (FIO) Data (type, presence, magnitude), Pathogen Data (type, presence, magnitude), system type (private, municipal, etc.), Detection Method, Outbreak Association, Sampling Period/Season.
3. “Pathway” data: Livestock/Agriculture Associated, Wild Animal Associated, Septic Tank/Sewage Associated, Precipitation/Climate Associated, Geology Associated, Source Associated, Local (Hydro)Geological Characteristics.

Pathogen records were extracted from all included studies, with each record representing analysis for one pathogen type or strain (regardless of actual presence). For example, if groundwater samples were analysed for both *E. coli* O157 and *Cryptosporidium* spp. within one study, two records were extracted. Study design was classified into four categories, including: repeat sampling (sources sampled more than once over extended time period), “snapshot” sampling (sources sampled on a “one off” basis), outbreak investigations and laboratory-based studies (studies whose primary objective was the development or refinement of a laboratory technique relating to enteric pathogen detection in environmental samples). Data pertaining to pathogen detection method were categorised into three groups, molecular diagnostics, microscopy and culture-based techniques. System type (private, community, municipal) was categorized based on explicit classification in included studies. Finally, pathogens were classified on the basis of being either potentially zoonotic or non-zoonotic, based on current CDC classification.

### 2.3 Statistical Analysis

All data analyses were performed with IBM SPSS Statistics 20. Chi-square tests were employed to examine significance between categorical variables, using R×L contingency tables and variable categories (dichotomous, ordinal). Continuous variables were subjected to one-way ANOVA (both weighted and un-weighted) analyses for both between- and within-group differences. One-way ANOVA was undertaken based upon prior Levene tests for the assessment of both between group and within group equality of variance. Bonferroni “post-hoc” multiple comparisons analysis was utilized after all ANOVA analyses to elucidate multi-criteria variance and exhibited association “directionality” (I–J) and to ensure highlighted differences (*p*<0.05) were not systematically dispersed among categories. Two-tailed Spearman’s’ rho was used to investigate correlation between continuous variables. Tests for variance and categorical homogeneity were undertaken prior to all analyses via both Welch and Brown-Forsythe robust tests for mean equality. A significance level of p<0.05 was used throughout the study [38].

## Results

### 3.1 Descriptive Analysis of Literature

A total of 55 published studies included extractable data, of which 23.6% (n = 13) were Canadian studies, while the remaining 76.4% (n = 42) were from the United States (Appendix S1). Included studies spanned the review time period, with a peak (n = 7) noted in 2002. Overall, 45.4% of studies (n = 25) contained both fecal indicator organism (FIO) and enteric pathogen detection data, while a further 29.1% (n = 16) contained enteric pathogen data only. The remaining 25.4% of studies (n = 14) solely reported on FIO detection. Of those studies that included extractable data pertaining to enteric pathogen detection (n = 41), 80.5% (n = 33) were from the US, while the remaining studies (n = 8) were from Canada. From these 41 studies, 102 extractable records were found (Tables 3 & 4); most were derived from US studies (90/102). All extracted enteric pathogen records were included for pooled-analyses.

**Table 3:** Enteric pathogen records from included US and Canadian studies (1990–2013) (n = 102); record number, percentage of total records, record number with specified pathogen present, percentage of records with specified pathogen present.

Pathogen included in study	N	%	Presence of pathogen	
			N	%
Bacteria				
VTEC^1^ (O157)	5	4.9	3	60
* Salmonella* spp.	5	4.9	4	80
EPEC^2^ (non O157)	4	3.9	4	100
* Campylobacter jejuni*	4	3.9	2	50
* Arcobacter* spp.	1	1	1	100
Shigella spp.	1	1	0	0
* Helicobacter* spp.	1	1	1	100
* Leptospira* spp.	1	1	0	0
* Yersinia* spp.	1	1	1	100
Protozoa				
* Cryptosporidium* spp.	9	8.8	6	66.7
* Giardia* spp.	10	9.8	3	30
* Naegleria fowleri*	1	1	1	100
**Virus**				
Enterovirus	14	13.7	14	100
Norovirus	12	11.8	9	75
Hepatitis A (HAV)	8	7.8	7	87.5
Rotavirus	7	6.9	4	57.1
Adenovirus	6	5.9	5	83.3
Human Enteric Viruses	5	4.9	3	60
Total Culturable Viruses	4	3.9	3	75
Reovirus	2	2	2	100
SRSV^3^	1	1	1	100
Total	102	100	74	72.5

1 Vero-toxigenic *Escherichia coli*.

2 Enteropathogenic *Escherichia coli*.

3 Small Round Structured Viruses.

**Table 4:** Summary statistics: a. Extracted Studies (n = 55), b. Extracted Records (n = 102), c. Extracted records associated with confirmed enteric pathogen presence (n = 74), d. Sampled well numbers (n = 52) and analysed groundwater sample numbers (n = 39).

Variable	Canada		United States	
	Total	Pathogen Present	Total	Pathogen Present
	N (%)	N (%)	N (%)	N (%)
**a.**				
**Supply Type (n = 54)**				
Municipal	4 (7.4)	3 (75)	11 (20.3)	9 (81.8)
Private	9 (16.6)	3 (33.3)	24 (44.4)	15 (62.5)
Community	0 (0)	-	5 (9.2)	4 (80)
Mixed	0 (0)	-	1 (1.8)	1 (100)
**Fecal Indicator Organisms (n = 39)**				
Total Coliforms	7 (17.9)	n/a	25 (54.1)	n/a
*E. coli*	10 (25.6)	n/a	24 (51.5)	n/a
Intestinal *enterococci*	5 (12.8)	n/a	11 (28.2)	n/a
Coliphage	2 (7.5)	n/a	6 (15.3)	n/a
*C. perfringens*	0 (0)	n/a	5 (12.8)	n/a
Heterotrophic Plate Counts	0 (0)	n/a	2 (7.5)	n/a
**Sample Season (n = 31)**				
Spring	0 (0)	-	4 (12.9)	2 (50)
Summer	2 (6.4)	1 (50)	8 (25.9)	2 (25)
Autumn	2 (6.4)	2 (100)	9 (29)	6 (66.7)
Winter	1 (3.2)	0 (0)	0 (0)	-
Year Round	0 (0)	-	5 (16.1)	4 (80)
**Pathogen Detection Method (n = 40)**				
Molecular Techniques	7 (17.5)	7 (100)	22 (55)	18 (81.8)
Microscopy	1 (2.5)	0 (0)	3 (7.5)	2 (66.7)
Culture-based	0 (0)	-	7 (17.5)	5 (71.4)
**b.**				
**Aquifer/Bedrock Type (n = 61)**				
Karstified	0 (0)	-	13 (21.3)	12 (92.3)
Unconsolidated	0 (0)	-	17 (27.8)	15 (88.2)
Fractured Bedrock	1 (1.6)	1 (100)	9 (14.7)	4 (44.4)
Un-fractured Bedrock	4 (6.5)	2 (50)	9 (14.7)	7 (77.7)
Diverse (Large Area)	0 (0)	-	8 (13.1)	7 (87.5)
**Study Design (n = 102)**				
Repeat Sampling	7 (6.8)	3 (42.8)	25 (24.5)	21 (84)
Snapshot Sampling	4 (3.9)	2 (50)	18 (17.6)	18 (100)
Outbreak Investigation	1 (1)	1 (100)	41 (40.2)	24 (58.5)
Laboratory-based	0 (0)	-	6 (5.8)	5 (83.3)
**c.**				
**Pathogen Type (n = 102)**				
Bacterial	2 (2)	2 (100)	20 (19.6)	12 (60)
Protozoan	6 (5.8)	2 (33)	15 (14.7)	9 (60)
Viral	4 (3.9)	2 (50)	55 (53.9)	46 (88.5)
**Pathogen Source (n = 74)**				
Agriculture/Livestock	n/a	4 (5.4)	n/a	5 (6.7)
Wild Animals	n/a	1 (1.3)	n/a	2 (2.7)
Septic tank/Sewage system	n/a	2 (2.7)	n/a	43 (58.1)
Surface Water Body	n/a	2 (2.7)	n/a	33 (44.6)
**Contamination Pathway (n = 74)**				
Precipitation	n/a	3	n/a	14 (18.9)
Geological	n/a	1	n/a	43 (58.1)
Source-specific	n/a	2	n/a	23 (31.1)
	**Canada**		**United States**	
	**Mean**	**Range**	**Mean**	**Range**
**d.**				
Well Number	15	1–1,208	160	1–5,520
Sample Number	41	1–1,208	225	1–5,520
Samples/well	2.73		1.4	

### 3.2 Fecal Indicator Organisms

In total, 39 studies reported FIO data related to six organisms. Based on the definition of FIO as “non-pathogenic, exclusively intestinal in habit and therefore exclusively fecal in origin if found outside the intestine” [39], total coliforms are not considered a FIO, however their use as indicator organisms remains widespread and thus are included here. A median of 2 FIOs were reported per study, up to a maximum of 5. *E. coli* was the FIO analysed most frequently, while heterotrophic plate counts were employed least often (Table 4a). Two-tailed Spearman’s’ rho was used to examine correlations between individual FIOs and the presence of enteric pathogens, in studies where both data were reported (Table 5). The presence of total coliforms, *E. coli*, intestinal Enterococci and coliphage (Male-specific (F+) and Somatic) were all strongly correlated with each other (p<0.01). No significant correlation between the total percentage of sample wells with enteric pathogens present (regardless of pathogen type) and the percentage of wells with fecal indicators present (individually or combined) was found. Weak (−0.7< r <0.7) negative correlations were found between pathogen contamination percentage (% of sampled wells with enteric pathogens present, irrespective of pathogen category) and total coliforms (% of sampled wells with total coliforms present) (r = −0.289, p = 0.034) and intestinal enterococci (r = −0.435, p = 0.006). A weak positive correlation between bacterial pathogen presence and *E. coli* (r = 0.636, p = 0.02). Both protozoan (r = 0.866, p = 0.026) and viral pathogen presence (r = −0.494, p = 0.023) were correlated with the presence of intestinal enterococci.

**Table 5:** Correlation matrix; fecal indictor organisms and total source contamination percentage (n = 25) (*Clostridium perfringens* and Heterotropic plate counts were excluded due to low record numbers).

			Total Coliforms	*E. coli*	Enterococci	Coliphage	% Contamination
Spearman’s rho	**Total Coliforms**	Correlation Coefficient	1	0.907**	0.923**	0.608**	0.289*
		Significance (2-tailed)	.	0	0	0.001	0.034
		N	54	41	38	28	54
	***E. coli***	Correlation Coefficient	0.907**	1	0.843**	0.627**	−0.161
		Significance (2-tailed)	0	.	0.001	0.001	0.269
		N	41	49	32	23	49
	**Enterococci**	Correlation Coefficient	0.923**	0.843**	1	0.601**	−0.435**
		Significance (2-tailed)	0	0		0.001	0.006
		N	38	32	39	29	39
	**Coliphage**	Correlation Coefficient	0.608**	0.627**	0.601**	1	−0.032
		Significance (2-tailed)	0.001	0.001	0.001	.	0.856
		N	28	23	29	30	30
	**% Contamination**	Correlation Coefficient	−0.289*	−0.161	−0.435**	−0.32	1
		Significance (2-tailed)	54	0.269	0.006	0.866	.
		N		49	39	30	62

**Correlation is significant at the 0.01 level (2-tailed).

*Correlation is significant at the 0.05 level (2-tailed).

### 3.3 Study Design & Pathogen Detection

Of 102 identified records, 74 (72.5%) reported confirmed pathogen presence, employing four main study designs. The most frequent study design focusing on enteric pathogen detection was (i) outbreak investigations (n = 18, 40%, 8.5 month mean duration), followed by (ii) repeat sampling based fieldwork (n = 14, 31.1%, 12.7 month mean duration), (iii) “snapshot” (i.e. “one-off”) sampling studies (n = 9, 20%, 12 month mean duration) and (iv) laboratory method-based studies (n = 2, 4.4%, 12 month mean duration). A significant association was noted between pathogen detection percentage (% of records in which enteric pathogens were positively found) and study design (χ^2^(3) = 7.749, p = 0.05), with a higher prevalence of pathogen detection found among “snapshot” type studies (Table 4b). Source contamination percentages ranged from 7.3% (lab-based studies) to 19.5% (outbreak investigations) of analysed groundwater samples, however, this was not found to be statistically significant. No correlation was found between either source or sample number analysed per study and source or sample contamination percentage; repeat sampling studies analysed a mean well and sample number of 23/130, equivalent mean sample numbers were 210/219, 25/269 and 219/263 for outbreak investigations, lab-based studies and “snapshot” studies, respectively. A significant difference was noted in relation to study design and analysed well number (F(3) = 3.468, p = 0.019), however, no significant difference was found between study type and sample number. Sample season was reported within 31 studies (Table 4a). While not statistically significant at the 0.05 level, enteric pathogens were more prevalent during autumn sampling events and year round repeat sampling studies. Study design and sampling season were significantly associated (χ^2^(12) = 34.594, p = 0.001); almost 60% of outbreak investigations took place during autumn, while 17.8%, 21% and 3.5% occurred during spring, summer and winter, respectively. Moreover, 100% of lab-based studies (5/5) comprised year round repeat sampling. Study design and enteric pathogen type were also associated (χ^2^(6) = 24.162, p<0.001); all (6/6) lab-based records were associated with waterborne enteric viruses, as were the majority (26/32) of repeat sampling studies. Outbreak investigations were split with respect to pathogen type (bacteria 14/42, protozoa 9/42, viral 19/42).

### 3.4 Pathogen Detection Method

The majority (72.5%) of pathogen detection was undertaken using molecular techniques (Table 4a). Microscopy was not utilised in any studies for the detection of bacterial pathogens (χ^2^(2) = 6.438, p = 0.04), which were most commonly analysed using molecular diagnostics (9/14). Similarly, molecular methods were favoured for the detection of protozoan pathogens (χ^2^(2) = 13.365, p = 0.001) (7/11). Studies reporting pathogen detection were approximately equally split between mono- (51.2%) and multi-pathogen (48.8%) detection. The presence of bacterial pathogens was analysed in 35.7% of studies, while protozoan and viral detection took place in 28.6% and 54.8% of studies, respectively. Two studies reported on all three enteric pathogen categories.

### 3.5 Sample Sources

As shown, 60% of studies (33/55) focused on private domestic wells, with a furter 24.2% (15/55) focusing on municipal groundwater systems (Table 4a). Further, 89.2% of samples were extracted from boreholes, while 7.8%, 2.0% and 1.0% were taken from spring sources, mixed sources and hand-dug wells, respectively. No statistical association was found between groundwater system type and groundwater source type at a 95% confidence level, however, pathogen presence and system type were associated at a 90% level (χ^2^(3) = 6.957, p = 0.074); a lower mean prevalence of enteric pathogen presence was found among community systems (Table 4a). In accordance with best practise guidelines, 87.3% of all groundwater samples were extracted “pre-treatment”, with a further 5.5% extracted both “pre-” and “post-treatment”. Just under half of the included studies (49.1%, n = 27) also included hydrogeological data including local hydrogeological setting (bedrock type, aquifer type, subsoil type/strata thickness, porosity, lithology, faulting, etc.).

The majority of records (69.6%, n = 71) were associated with “non-outbreak” conditions, while the remaining were instigated as a direct result of a confirmed waterborne outbreak. A significant association was found between the prevalence of outbreak-related studies and groundwater system type (χ^2^(3) = 9.644, p = 0.022); community systems were significantly more likely to be associated with waterborne epidemic conditions. No association was found between the instance of waterborne outbreaks and groundwater source type, nor between outbreak-related studies and pathogen strain or type. Viral presence was significantly higher than either bacterial or protozoan presence (χ^2^(2) = 9.505, p = 0.009); overall, viral pathogens were found in 85.7% of studies in which viral detection methods were employed (Table 4c) (regardless of outbreak/non-outbreak conditions).

### 3.6 Hazard/Pathogen Source

Data extraction was carried out for potential pathogen sources (agriculture/livestock, wild animals, septic tanks/sewage systems, surface water bodies) within all waterborne pathogen records. Data were extracted based upon being a confirmed (e.g. use of tracer study) or likely (e.g. population cohort study) pathogen source. Septic tanks/sewage systems were the most frequently reported confirmed or likely pathogen source (Table 4c). No statistically significant association was found to exist between pathogen presence (Yes/No) and any specific potential pathogen source. Overall, 52% of records (53/102) referred to potentially zoonotic pathogens, which were over 5 times more likely to be present when adjacent agricultural activities were recorded (OR = 5.465, 95% CI 1.133, 26.361; χ^2^(1) = 5.362, p = 0.021) (Table 4c). Additionally, potentially zoonotic pathogens were over twice as likely to be present where the groundwater source was located adjacently to a surface water body (OR = 2.739, 95% CI 1.214, 6.178; χ^2^(1) = 6.031, p = 0.017). Conversely, potentially zoonotic pathogens were approximately 3 times less likely to be detected where septic tanks and/or municipal sewage systems were noted as the primary local pathogen source (OR = 0.337, 95% CI 0.145, 0.784; χ^2^(1) = 6.574, p = 0.014). Potentially zoonotic pathogens were more likely to be detected in shallow hand-dug and spring sources than deeper boreholes (χ^2^(3) = 11.751, p = 0.008). Pathogens were categorised based upon pathogen type (bacterial, protozoan, viral or multiple) and similar analysis undertaken with respect to pathogen presence and potential pathogen source. Only one significant association resulted, with bacterial presence 75% more likely in areas where livestock presence was recorded (OR = 1.75, 95% CI 1.112, 2.775; χ^2^(1) = 4.714, p = 0.03).

### 3.7 Contamination Pathways

Three potential contamination pathways were identified during data extraction, including precipitation patterns (leading to contamination ingress at the wellhead or rapid subsurface infiltration), geological pathways (increased contaminant transmissivity via unconsolidated/fractured aquifer materials or increased runoff coefficients) and inadequate source design/construction (e.g. uncovered wellhead, cracked jointing, absence of sanitary seal, etc.) (Table 4c). It is important to note that while these pathways are typically interconnected, with the presence of one typically amplifying the effects of others, however, the purpose of the current review is only to report on patterns within the included literature. No association was found between pathogen presence and either increased precipitation or geological pathways, however, inadequately designed or constructed wells were found to have pathogens present in 50% more records than adequately designed sources as noted by authors (OR = 1.51, 95% CI 1.283, 1.777; χ^2^(1) = 12.531, p<0.001). Overall, 59.8% of records included data pertaining to aquifer/bedrock type (n = 61) (Table 4c).

No association was noted between the presence of bacterial pathogens and precipitation patterns or geological pathways; however, 100% of sources with confirmed bacterial pathogen presence were noted as being poorly designed/constructed, indicating “source-specific” contaminant pathways (OR = 2.333, 95% CI 1.274, 4.272; χ^2^(1) = 7.184, p = 0.007). Likewise, inadequately designed/constructed wells were almost 3 times more likely to have enteric protozoa present (OR = 2.75, 95% CI 1.258, 6.01; χ^2^(1) = 9.545, p = 0.004). Viral pathogen presence was less likely to be associated with precipitation patterns (OR = 0.091, 95% CI 0.016, 0.509; χ^2^(1) = 9.722, p = 0.002), with no association noted regarding well design/construction or local geological characteristics.

### 3.8 Study Location

Canadian and US groundwater contamination studies over the past two decades were similar based on study years, use of and FIO types tested, frequency of enteric pathogen sampling and analysis, types of wells and geological areas sampled, in addition to utilised sampling and pathogen detection methods (Table 4a–b). No significant mean differences were noted between Canadian and US studies with respect to either enteric pathogen presence (well %, sample %) or pathogen presence category (bacteria/protozoa/virus). Canadian studies included in data extraction were derived from 5 (50%) provinces and 1 territory (Yukon), while US studies included data from 21 (42%) mainland states and 5 large multi-state (>10) studies. Ontario (n = 5) and Wisconsin (n = 8) were the most frequently represented Canadian province and US state, respectively.

Some notable differences were found with respect to study country; while only 23% (n = 3) of Canadian studies noted local (hydro)geological characteristics. These parameters were reported in 57% of US studies (24/42) (χ^2^(1) = 4.61, p = 0.032). Included Canadian studies differed from US studies with respect to study design (χ^2^(3) = 8.386, p = 0.039). For example, 100% of lab-based study records (6/6) were derived from the US, while a higher proportion of repeat sampling records were from Canadian studies (Table 4b). US studies were associated with both higher mean sampled well numbers (t = 4.369, p<0.001) and higher mean sample numbers (t = 5.389, p<0.001), while a higher mean number of samples per well was attributed to Canadian studies (Table 4d). Studied pathogen category differed with respect to study location (χ^2^(2) = 7.278, p = 0.026); while just over 12% of extracted pathogen records were derived from Canadian studies, 29% (n = 6) of protozoan records were associated with Canada.

Tests of independence were used to ascertain whether specific contaminant sources or pathways were equally reported based on study location. Both agricultural sources (point and diffuse) and septic tanks/municipal sewage treatment reporting differed significantly (Table 4c). While agricultural livestock were cited as potential pathogen sources in 34% (n = 4) of Canadian records, only 6% of US records (n = 5) did likewise (χ^2^(1) = 19.153, p<0.001). Conversely, while only 16% of Canadian records mentioned septic tanks/municipal sewage systems with respect to pathogen presence, 58% of US studies (n = 43) reported this link (χ^2^(1) = 8.289, p = 0.008). Geology was cited as a likely contaminant pathway in only 8.3% (n = 1) of Canadian records and in 58% (n = 43) of US records (χ^2^(1) = 11.376, p = 0.001).

## Discussion

A review of groundwater pathogen contamination studies in Canada and the US was conducted, locating 102 enteric pathogen records from 55 studies. The majority of studies were from the United States. While almost 24% of studies were Canadian, only 12% of enteric pathogen records were from Canadian studies. Typically, US studies were more likely to include “multi-pathogen” data (>1 enteric pathogen per study) and therefore >1 record, while Canadian studies were more likely to include FIO data with no accompanying enteric pathogen data. There is a paucity of studies focusing on direct pathogen detection in groundwater systems in both countries. Given concerns regarding the use of FIOs as surrogate organisms [40]–[42], the suitability of FIOs for use in the regulatory standards for protection of water quality is a particular concern. Overall, 38.5% of Canadian studies contained FIO data in the absence of enteric pathogen data, potentially resulting in significant under-estimates of enteric pathogen presence and/or magnitude. Future work should focus on direct detection of enteric pathogens; this data may be used to improve microbial risk assessment efforts via significantly improved exposure assessment.

Just over 42% of extracted records in this review were for private domestic wells. Currently 34–45% of groundwater users in Canada and approximately 33% of groundwater users in the US employ a private water source [5], [8]. There is a lack of enteric pathogen data from municipal supplies, reflecting a potentially critical knowledge gap. The inclusion of municipal groundwater supplies in future North American groundwater sampling studies would help inform this identified gap.

In Canada, the focus within scientific studies in the reviewed literature has been on protozoan detection in groundwater, while in the US, it has been on bacterial and viral detection, with viruses being the most commonly studied pathogen in this review. Pathogenic viruses are a particular concern in groundwater systems as their size enables them to evade natural attenuation processes and enter the aquifer [43], [44].

No correlation was found between viral presence and either total coliforms or *E. coli,* the two most frequently employed FIOs for groundwater system surveillance. Moreover no association between coliphage detection and viral presence was observed in this review. A 1-year study of 12 municipal wells in Quebec, Canada reported similar results [45]. As previously noted by Borchardt *et al*. [44], the majority of groundwater-associated municipal systems in the US produce water without disinfection, populations served by these systems are therefore potentially exposed to waterborne viruses and consequent health risks. The limited value of FIOs in predicting the presence of enteric viruses in groundwater highlights the need for direct enteric viral detection at groundwater sources used for drinking water [46]–[48]. The lack of significance between viral presence and source design, in concurrence with an association between viral presence and precipitation patterns, suggests a generalised mode of contamination associated with viral pathogens [13].

Geology was rarely recorded or presented as a potential contaminant pathway in Canadian studies, while 60% of US studies stated that local geological conditions were a potential cause for concern. Awareness of local geological and hydrogeological characteristics is critical in understanding likely contamination mechanisms or pathways, resource management and remediation and, by extension, human health risk assessment [49]–[50]. Future research relating to microbial contamination of North American groundwater systems, that adequately describes geological and hydrogeological characteristics of the groundwater source will be more useful for informing our understanding of public health risk. Future work related to the assessment of treatment requirements in geologically vulnerable regions based on the likelihood of pathogen presence would be particularly useful.

Bacterial pathogens accounted for 22.6% of records; all studies identified poorly designed or constructed well sources as a likely pathway. In contrast to the viral pathogen findings, this suggests localized, source-specific contamination as a predominant ingress mechanism e.g. groundwater contamination occurring at source due to poor wellhead protection as opposed to generalised contamination of all or part of the aquifer [13], [51]. This is further reflected by the lack of association between bacterial presence and local hydrogeological characteristics.

The increased likelihood of potential zoonotic pathogen presence in agricultural areas was anticipated; low travel times in the subsurface make it unlikely to find these pathogens in areas without animals (livestock or wildlife) [18], [22], [52]. Our review also found bacterial pathogen presence more likely than either viral or protozoan presence in rural areas with livestock present. Potentially zoonotic pathogens were also more likely to be present where the sampled well was located in a surface water catchment, suggesting the potential for direct (and potentially rapid) groundwater-surface water interaction as a contamination mechanism. Potentially zoonotic pathogens were significantly less likely to be present in urban areas. Studies outside North America have previously reported pathogens as more likely present in shallow hand-dug and spring sources; for example, Hynds *et al*. [13] found a higher prevalence of pathogen presence in shallow groundwater sources in the Republic of Ireland. Hand-dug wells and springs are typically associated with inferior design and construction, leading to an increased likelihood of direct pathogen ingress at the wellhead [51], [53]–[55]. It is important to note that both study design and reporting are likely to introduce bias in these results, for example, studies tended to focus on groundwater systems in rural/agricultural areas, while few examined urban systems. Accordingly, interpretation of these results may not adequately reflect hazard sources or contamination pathways, being are based solely upon available data.

Much of North America, particularly the United States, is characterised by unconsolidated aquifers [7]. Accordingly, unprotected shallow groundwater systems are regularly used, particularly for domestic supply. Wallender *et al*. [16] have reported that 26.2% of waterborne outbreaks from 1971 to 2008 were associated with karst limestone bedrock. Consumers and water managers in these areas would benefit from a clear understanding of the potential contamination risks, and consequent human health burden posed by using untreated shallow systems, and intervention options that are most effective. These findings may be useful for informing future groundwater surveillance/monitoring study design with respect to the most appropriate pathogen (category/species) to include for analysis, and where to focus the monitoring efforts. Moreover, where a human enteric disease outbreak is suspected, these criteria may be used to elucidate the potential infection etiology.

Based on this review, we note an inconsistency between studied groundwater pathogen (category) and reported groundwater-related human health burden in North America. A knowledge gap exists with respect to information on both prevalence and concentration of pathogen contamination of groundwater systems, particularly enteric bacteria. For example, it has been reported that 11% of waterborne outbreaks were caused by *Escherichia coli* O157 (VTEC) as a confirmed etiological agent [56]; however, only 4.9% of pathogen records in this review relate to VTEC (Table 3).

Little efficacy was found with respect to the predictive ability of FIOs and protozoan or viral contamination of groundwater systems. The lack of association between protozoan presence and local hydrogeological characteristics (as for bacterial pathogens) reinforces the importance of rapid by-pass mechanisms [13], with poorly designed wells as much as three times more likely to have protozoan pathogens present. Consequently, correctly implemented source management, including appropriate well location, design, construction and maintenance, in concurrence with periodic direct pathogen analysis is of paramount importance. This is particularly significant in light of the resistance of *Cryptosporidium* spp. to conventional chlorination [57]. It is considered that an increase in direct pathogen data analysis and reporting would prove useful for future risk management and communication strategies to inform intervention decisions and source water protection initiatives. For example, Morris & Foster [58] have noted that protozoan pathogens are commonly believed to be absent from “true” groundwater due to their size; however, with better data on groundwater contamination this assumption could be evaluated to further inform risk identification and mitigation strategies.

Results and conclusions presented here are based on the extracted data from published studies and are subject to the inherent biases of the original study objectives, designs and what was reported. Few Canadian studies referenced local hydrogeological characteristics as a potential contamination pathway when reporting on contamination events or groundwater-associated outbreaks, representing a limitation in study reporting. This lack of reporting may impact subsequent analyses of contamination pathways, for example, findings may reflect a lack of reporting or examination of these factors in a rigorous manner. Furthermore, only one included study occurred in winter, representing a potential limitation.

Study design differed significantly between US and Canadian studies. The majority of lab-based studies and outbreak investigations were in the US, while a higher proportion of repeat sampling records were from Canada. Repeat sampling studies integrated low well numbers with multiple samples from each well (mean – 6 samples). Outbreak investigations and “snapshot” studies comprised significantly higher well numbers, with wells typically sampled once. Outbreak investigations are an essential epidemiological tool; however, they are reactive and therefore not suitable for assessing endemic groundwater risks. Numerous studies report that multiple samples are necessary to positively determine contamination of a groundwater source [59]–[60]. Accordingly, repeat sampling studies are more likely to reflect contamination rates in Canadian and US groundwater systems; however few were identified in this review.

Enteric pathogens were present in 72.5% of records (74/102) and 16.1% of wells. Upon exclusion of outbreak investigation data, 77.1% of records (54/70) and 14.7% of wells (n = 1855/12,616; Std. Dev. 20.5%) were positive for at least one enteric pathogen. These figures may provide a useful foundation for endemic groundwater-associated exposure assessment, until more direct enteric pathogen data become available.

The lack of association between the incidence of outbreak investigations and municipal and private groundwater systems was not anticipated; however, as the majority of groundwater sampling was undertaken pre-treatment, this does not accurately reflect the public health burden posed by specific system types (e.g. many private sources remain untreated or poorly treated, thus raw water samples may be more reflective of drinking water quality). Large municipal systems are more likely to be adequately maintained and appropriately treated [24]–[25]. Smaller community systems were represented by a significantly higher proportion of outbreak investigations. A recent review of waterborne outbreaks by Hrudey [61] found that all, with the exception of one (Milwaukee, 1993), occurred in community systems. Furthermore, Craun *et al*. [62] have reported on ten cryptosporidiosis outbreaks in the US directly attributable to community water systems. Accordingly, the characteristics of a robust groundwater supply should include an understanding of the source, delivery of adequate treatment, adequate system monitoring and effective response to monitoring results when required, particularly in the case of private and community systems where the primary responsibility for source quality lies with owners and users [63].

Our review has highlighted a number of knowledge gaps and requirements relating to contamination of Canadian and US groundwater systems over the past two decades. Improved endemic risk assessment will be possible with increased direct enteric pathogen detection and reporting. Repeat-sampling studies represent a priority for the design of future studies for the collation of these data, as they are considered more representative with respect to human endemic risk. More data are required for groundwater-associated municipal systems, both pre- and post-treatment, as currently these systems are not adequately represented in the literature. Additionally, more data are required for bacterial pathogens, as these microorganisms are associated with a large public health burden. These data would also provide a foundation for improved risk management and communication via increased awareness and understanding among well owners, well users, system operators and policy makers.

## Conclusions

- Within the literature the four study designs that were identified were of a proactive (pre-waterborne illness event) or reactive (post-waterborne illness event) nature, with the proactive designs being more suitable for background characterisation. Pooled analyses of reported data found that enteric pathogens were present in approximately 15% of sampled wells; this may be useful for future microbial risk assessments of human health relating to Canadian and US groundwater systems, although it should only be employed in the absence of area-specific data, and then, only with caution due to inherent limitations associated with the current study (e.g. reporting biases associated with amalgamation of differing study designs, lack of data relating to specific hydrogeological settings, lack of data associated with winter sampling and weather events, etc.). Accordingly, the authors recommend future research be focused on addressing this current lack of temporal and spatial data.
- It is critically important to account for study design when presenting and interpreting results of a groundwater study, given that groundwater is not similar to surface water and many confounding factors will influence the presence of pathogens in one system versus another, as well as the occurrence of an associated outbreak.
- Few studies have been published on pathogen presence in municipal groundwater systems. By addressing this knowledge gap, evidence would be developed to help support local decision-making around appropriate treatment or other interventions to further promote the safety of the drinking water supply. It is recommended that future health-related studies, particularly those associated with sporadic waterborne infection and quantitative health risk assessments, focus on direct enteric pathogen detection where possible, given the limited efficacy of fecal indicator organisms for enteric pathogen presence in groundwater systems. These data will aid more focused, pathogen-specific exposure assessment and hazard characterisation, therefore providing more data for evidence-based groundwater and human-health protection measures.
- Local (hydro)geology is an important risk factor in the likelihood of pathogen presence in a groundwater supply and was not reported in a substantial proportion of study records included in this review. The availability and reporting of these data could be used in combination with land-use, source type and seasonality to more accurately inform outbreak investigators, epidemiologists and sanitary engineers.
- Bacterial and protozoan contamination appear to be associated with localised contamination mechanisms (well-specific), lending credence to the concept of “consumer control” (education of private well users may directly affect contamination rates and health burden). Viral contamination may be more generalised (aquifer scale) but intervention at the source could be targeted to address all enteric pathogens (e.g. ultraviolet disinfection).
- Our findings further reflect the importance of frequent inspection and maintenance of both private and municipal groundwater supplies in Canada and the US. Additionally, it is important that potential system vulnerabilities including climate, local hydrogeological setting and adjacent hazard sources are considered during study design.

## Supporting Information

Appendix S1 **55 included studies in the review with extractable data.**
(DOCX)

Checklist S1 **PRISMA guideline checklist for reporting systematic reviews.**
(DOC)
