# Supplementary material for: Contamination of Groundwater Systems in the US and Canada by Enteric Pathogens, 1990–2013: A Review and Pooled-Analysis
Source: PLoS One. 2014 May 7;9(5):e93301. doi: 10.1371/journal.pone.0093301 (PMC4012953; doi:10.1371/journal.pone.0093301)
Supplement: Appendix S1 — 55 included studies in the review with extractable data. (DOCX) [file pone.0093301.s001.docx]

**Appendix 1. Fifty-five studies included after review procedure**

[Abbaszadegan M, Lechevallier M, Gerba C](http://www.refworks.com/refworks2/default.aspx?r=references%7CMainLayout::init) (2003) Occurence of viruses in US groundwaters. [*AWWA*](http://www.refworks.com/refworks2/default.aspx?r=references%7CMainLayout::init). **95**(9)

[Abbaszadegan M](http://www.refworks.com/refworks2/default.aspx?r=references%7CMainLayout::init), [Stewart P](http://www.refworks.com/refworks2/default.aspx?r=references%7CMainLayout::init), [LeChevallier M](http://www.refworks.com/refworks2/default.aspx?r=references%7CMainLayout::init) (1999) A strategy for detection of viruses in groundwater by PCR. [*Appl. Environ. Microbiol*.](http://www.refworks.com/refworks2/default.aspx?r=references%7CMainLayout::init) **65**(2):444-449

[Abbaszadegan M](http://www.refworks.com/refworks2/default.aspx?r=references%7CMainLayout::init), [Huber MS](http://www.refworks.com/refworks2/default.aspx?r=references%7CMainLayout::init), [Gerba CP,](http://www.refworks.com/refworks2/default.aspx?r=references%7CMainLayout::init) [Pepper IL](http://www.refworks.com/refworks2/default.aspx?r=references%7CMainLayout::init) (1993) Detection of enteroviruses in groundwater with the polymerase chain reaction. [*Appl. Environ. Microbiol.*](http://www.refworks.com/refworks2/default.aspx?r=references%7CMainLayout::init) **59**(5):1318-1324

[Anderson AD](http://www.refworks.com/refworks2/default.aspx?r=references%7CMainLayout::init), [Heryford AG](http://www.refworks.com/refworks2/default.aspx?r=references%7CMainLayout::init), [Sarisky JP](http://www.refworks.com/refworks2/default.aspx?r=references%7CMainLayout::init), [Higgins C](http://www.refworks.com/refworks2/default.aspx?r=references%7CMainLayout::init), [Monroe SS](http://www.refworks.com/refworks2/default.aspx?r=references%7CMainLayout::init), et al (2003) A waterborne outbreak of Norwalk-like virus among snowmobilers-Wyoming, 2001. [*Jour. Infect. Dis.*](http://www.refworks.com/refworks2/default.aspx?r=references%7CMainLayout::init) **187**(2):303-306

[Anderson ME](http://www.refworks.com/refworks2/default.aspx?r=references%7CMainLayout::init), [Sobsey MD](http://www.refworks.com/refworks2/default.aspx?r=references%7CMainLayout::init) (2006) Detection and occurrence of antimicrobially resistant *E. coli* in groundwater on or near swine farms in eastern North Carolina. [*Water Sci. Technol.*](http://www.refworks.com/refworks2/default.aspx?r=references%7CMainLayout::init) **54**(3):211-218

[Angulo FJ](http://www.refworks.com/refworks2/default.aspx?r=references%7CMainLayout::init), [Tippen S](http://www.refworks.com/refworks2/default.aspx?r=references%7CMainLayout::init), [Sharp DJ](http://www.refworks.com/refworks2/default.aspx?r=references%7CMainLayout::init), [Payne BJ](http://www.refworks.com/refworks2/default.aspx?r=references%7CMainLayout::init), [Collier C](http://www.refworks.com/refworks2/default.aspx?r=references%7CMainLayout::init), et al (1997) A community waterborne outbreak of salmonellosis and the effectiveness of a boil water order. [*Am. Jour. Public Health*](http://www.refworks.com/refworks2/default.aspx?r=references%7CMainLayout::init). **87**(4):580-584

[Beller M](http://www.refworks.com/refworks2/default.aspx?r=references%7CMainLayout::init), [Ellis A](http://www.refworks.com/refworks2/default.aspx?r=references%7CMainLayout::init), [Lee SH](http://www.refworks.com/refworks2/default.aspx?r=references%7CMainLayout::init), [Drebot MA](http://www.refworks.com/refworks2/default.aspx?r=references%7CMainLayout::init), [Jenkerson SA](http://www.refworks.com/refworks2/default.aspx?r=references%7CMainLayout::init), et al (1997) Outbreak of viral gastroenteritis due to a contaminated well. International consequences. [*JAMA*](http://www.refworks.com/refworks2/default.aspx?r=references%7CMainLayout::init). **278**(7):563-568

[Betancourt WQ](http://www.refworks.com/refworks2/default.aspx?r=references%7CMainLayout::init), [Rose JB](http://www.refworks.com/refworks2/default.aspx?r=references%7CMainLayout::init) (2005) Microbiological assessment of ambient waters and proposed water sources for restoration of a Florida wetland. [*Jour. Water. Health*.](http://www.refworks.com/refworks2/default.aspx?r=references%7CMainLayout::init) **3**(2):89-100

[Bickford T, Lindsey B, Beaver M](http://www.refworks.com/refworks2/default.aspx?r=references%7CMainLayout::init) (1996) Bacteriological quality of groundwater used for groundwater supply. [USGS Report](http://www.refworks.com/refworks2/default.aspx?r=references%7CMainLayout::init) 1996 Available: <http://pa.water.usgs.gov/reports/wrir_96-4212/report.html> Accessed 2014 April 4

[Blair B](http://www.refworks.com/refworks2/default.aspx?r=references%7CMainLayout::init), [Sarkar P](http://www.refworks.com/refworks2/default.aspx?r=references%7CMainLayout::init), [Bright KR](http://www.refworks.com/refworks2/default.aspx?r=references%7CMainLayout::init), [Marciano-Cabral F](http://www.refworks.com/refworks2/default.aspx?r=references%7CMainLayout::init), [Gerba CP](http://www.refworks.com/refworks2/default.aspx?r=references%7CMainLayout::init) (2008) *Naegleria fowleri* in well water. [*Emerg. Infect. Dis*.](http://www.refworks.com/refworks2/default.aspx?r=references%7CMainLayout::init) **14**(9):1499-1501

[Bopp DJ](http://www.refworks.com/refworks2/default.aspx?r=references%7CMainLayout::init), [Sauders BD](http://www.refworks.com/refworks2/default.aspx?r=references%7CMainLayout::init), [Waring AL](http://www.refworks.com/refworks2/default.aspx?r=references%7CMainLayout::init), [Ackelsberg J](http://www.refworks.com/refworks2/default.aspx?r=references%7CMainLayout::init), [Dumas N](http://www.refworks.com/refworks2/default.aspx?r=references%7CMainLayout::init) et al (2003) Detection, isolation, and molecular subtyping of *Escherichia coli* O157:H7 and *Campylobacter jejuni* associated with a large waterborne outbreak. [*Jour. Clin. Microbiol*.](http://www.refworks.com/refworks2/default.aspx?r=references%7CMainLayout::init) **41**(1):174-180

[Borchardt MA](http://www.refworks.com/refworks2/default.aspx?r=references%7CMainLayout::init), [Spencer SK](http://www.refworks.com/refworks2/default.aspx?r=references%7CMainLayout::init), [Kieke BA](http://www.refworks.com/refworks2/default.aspx?r=references%7CMainLayout::init), [Lambertini E](http://www.refworks.com/refworks2/default.aspx?r=references%7CMainLayout::init), [Loge FJ](http://www.refworks.com/refworks2/default.aspx?r=references%7CMainLayout::init) (2012) Viruses in nondisinfected drinking water from municipal wells and community incidence of acute gastrointestinal illness. [*Environ. Health Perspect*.](http://www.refworks.com/refworks2/default.aspx?r=references%7CMainLayout::init) **120**(9):1272-1279

[Borchardt MA](http://www.refworks.com/refworks2/default.aspx?r=references%7CMainLayout::init), [Bradbury KR](http://www.refworks.com/refworks2/default.aspx?r=references%7CMainLayout::init), Alexander EC, [Kolberg RJ](http://www.refworks.com/refworks2/default.aspx?r=references%7CMainLayout::init), [Alexander SC](http://www.refworks.com/refworks2/default.aspx?r=references%7CMainLayout::init) et al(2011) Norovirus outbreak caused by a new septic system in a dolomite aquifer. [*Ground Water*](http://www.refworks.com/refworks2/default.aspx?r=references%7CMainLayout::init). **49**(1):85-97

[Borchardt MA](http://www.refworks.com/refworks2/default.aspx?r=references%7CMainLayout::init), [Bradbury KR](http://www.refworks.com/refworks2/default.aspx?r=references%7CMainLayout::init), [Gotkowitz MB](http://www.refworks.com/refworks2/default.aspx?r=references%7CMainLayout::init), [,](http://www.refworks.com/refworks2/default.aspx?r=references%7CMainLayout::init) [Parker BL](http://www.refworks.com/refworks2/default.aspx?r=references%7CMainLayout::init) (2007) Human enteric viruses in groundwater from a confined bedrock aquifer. [*Environ. Sci. Technol.*](http://www.refworks.com/refworks2/default.aspx?r=references%7CMainLayout::init) **41**(18):6606-6612

[Borchardt MA](http://www.refworks.com/refworks2/default.aspx?r=references%7CMainLayout::init), [Haas NL](http://www.refworks.com/refworks2/default.aspx?r=references%7CMainLayout::init), [Hunt RJ](http://www.refworks.com/refworks2/default.aspx?r=references%7CMainLayout::init) (2004) Vulnerability of drinking-water wells in La Crosse, Wisconsin, to enteric-virus contamination from surface water contributions. [*Appl. Environ. Microbiol.*](http://www.refworks.com/refworks2/default.aspx?r=references%7CMainLayout::init) **70**(10):5937-5946

[Borchardt MA](http://www.refworks.com/refworks2/default.aspx?r=references%7CMainLayout::init), [Chyou PH](http://www.refworks.com/refworks2/default.aspx?r=references%7CMainLayout::init), [DeVries EO](http://www.refworks.com/refworks2/default.aspx?r=references%7CMainLayout::init), [Belongia EA](http://www.refworks.com/refworks2/default.aspx?r=references%7CMainLayout::init) (2003) Septic system density and infectious diarrhea in a defined population of children. [*Environ. Health Perspect*.](http://www.refworks.com/refworks2/default.aspx?r=references%7CMainLayout::init) **111**(5):742-748

[Bruce Gray Owen Sound Health Unit](http://www.refworks.com/refworks2/default.aspx?r=references%7CMainLayout::init) (2000) The investigative report of the Walkerton outbreak of waterborne gastroenteritis. Report/2000 Available: <http://water.sesep.drexel.edu/outbreaks/WalkertonReportOct2000/REPORT_Oct00.PDF>

Accessed 2014 April 4

Budo-Amoako E, Greenwood SJ, Dixon BR, Barkema HW, McClure JT (2012) *Giardia* and *Cryptosporidium* on dairy farms and the role these farms may play in contaminating water sources in Prince Edward Island, Canada. [*Jour. Vet. Intern. Med*.](http://www.refworks.com/refworks2/default.aspx?r=references%7CMainLayout::init) **26**(3):668-673

Budo-Amoako E, Greenwood SJ, Dixon BR, Barkema HW, McClure JT (2012) Occurrence of *Cryptosporidium* and *Giardia* on beef farms and water sources within the vicinity of the farms on Prince Edward Island, Canada. [*Vet. Parasitol*.](http://www.refworks.com/refworks2/default.aspx?r=references%7CMainLayout::init) **184**(1):1-9

[Bunnell JE](http://www.refworks.com/refworks2/default.aspx?r=references%7CMainLayout::init), [Tatu CA](http://www.refworks.com/refworks2/default.aspx?r=references%7CMainLayout::init), [Bushon RN](http://www.refworks.com/refworks2/default.aspx?r=references%7CMainLayout::init), [Stoeckel DM](http://www.refworks.com/refworks2/default.aspx?r=references%7CMainLayout::init), [Brady AM](http://www.refworks.com/refworks2/default.aspx?r=references%7CMainLayout::init) et al(2006) Possible linkages between lignite aquifers, pathogenic microbes, and renal pelvic cancer in northwestern Louisiana, USA. [*Environ. Geochem. Health*](http://www.refworks.com/refworks2/default.aspx?r=references%7CMainLayout::init)*.* **28**(6):577-587

[Charatan F](http://www.refworks.com/refworks2/default.aspx?r=references%7CMainLayout::init) (1999) New York outbreak of *E. coli* poisoning affects 1000 and kills two. [*BMJ*](http://www.refworks.com/refworks2/default.aspx?r=references%7CMainLayout::init). **319**(7214)

[Daly ER](http://www.refworks.com/refworks2/default.aspx?r=references%7CMainLayout::init), [Roy SJ](http://www.refworks.com/refworks2/default.aspx?r=references%7CMainLayout::init), [Blaney DD](http://www.refworks.com/refworks2/default.aspx?r=references%7CMainLayout::init), [Manning JS](http://www.refworks.com/refworks2/default.aspx?r=references%7CMainLayout::init), [Hill VR](http://www.refworks.com/refworks2/default.aspx?r=references%7CMainLayout::init) et al (2010) Outbreak of giardiasis associated with a community drinking-water source. [*Epidemiol. Infect*.](http://www.refworks.com/refworks2/default.aspx?r=references%7CMainLayout::init) **138**(4):491-500

[Dworkin MS, Goldman DP, Wells TG, Kobayashi JM, Herwaldt BL](http://www.refworks.com/refworks2/default.aspx?r=references%7CMainLayout::init) (1996) Cryptosporidiosis in Washington State: an outbreak associated with well water. *Jour. Infect. Dis*. **174**:1372-6

Fitzgerald D, Chanasyk DS, Neilson RD, Keily D, Audette R (2001) Farm Well Water Quality in Alberta. *Water Qual. Res. Jour. Canada.* **36**(3):565-588

[Femmer S](http://www.refworks.com/refworks2/default.aspx?r=references%7CMainLayout::init) (1998) Microbiological and Chemical Quality of Ground Water used as a Source of Public Suplly in Southern Missouri. [USGS Report](http://www.refworks.com/refworks2/default.aspx?r=references%7CMainLayout::init) 1998 Available: <http://pubs.er.usgs.gov/publication/wri20004260>

Accessed 2014 April 4

[Fong TT](http://www.refworks.com/refworks2/default.aspx?r=references%7CMainLayout::init), [Mansfield LS](http://www.refworks.com/refworks2/default.aspx?r=references%7CMainLayout::init), [Wilson DL](http://www.refworks.com/refworks2/default.aspx?r=references%7CMainLayout::init), [Schwab DJ](http://www.refworks.com/refworks2/default.aspx?r=references%7CMainLayout::init), [Molloy SL](http://www.refworks.com/refworks2/default.aspx?r=references%7CMainLayout::init) [et](http://www.refworks.com/refworks2/default.aspx?r=references%7CMainLayout::init) al (2007) Massive microbiological groundwater contamination associated with a waterborne outbreak in Lake Erie, South Bass Island, Ohio. [*Environ. Health Perspect*.](http://www.refworks.com/refworks2/default.aspx?r=references%7CMainLayout::init) **115**(6):856-864

[Fout GS](http://www.refworks.com/refworks2/default.aspx?r=references%7CMainLayout::init), [Martinson BC](http://www.refworks.com/refworks2/default.aspx?r=references%7CMainLayout::init), [Moyer MW](http://www.refworks.com/refworks2/default.aspx?r=references%7CMainLayout::init), [Dahling DR](http://www.refworks.com/refworks2/default.aspx?r=references%7CMainLayout::init) (2003) A multiplex reverse transcription-PCR method for detection of human enteric viruses in groundwater. [*Appl. Environ. Microbiol*.](http://www.refworks.com/refworks2/default.aspx?r=references%7CMainLayout::init) **69**(6):3158-3164

[Francy DS](http://www.refworks.com/refworks2/default.aspx?r=references%7CMainLayout::init), Bushon RN, Stopar J, [Lozano EJ](http://www.refworks.com/refworks2/default.aspx?r=references%7CMainLayout::init), [Fout GS](http://www.refworks.com/refworks2/default.aspx?r=references%7CMainLayout::init) (2004) Environmental factors and chemical and microbiological water-quality constituents related to the presence of enteric viruses in ground water from small public water supplies in southeastern Michigan. [USGS Report](http://www.refworks.com/refworks2/default.aspx?r=references%7CMainLayout::init) 2004 Available: <http://pubs.usgs.gov/sir/2004/5219/> Accessed 2014 April 4

[Gonzales TR](http://www.refworks.com/refworks2/default.aspx?r=references%7CMainLayout::init) (2008) The effects that well depth and wellhead protection have on bacterial contamination of private water wells in the Estes Park Valley, Colorado. [*Jour. Environ. Health*](http://www.refworks.com/refworks2/default.aspx?r=references%7CMainLayout::init). **71**(5):17-23

[Goss MJ, Barry DA, Rudolph DL](http://www.refworks.com/refworks2/default.aspx?r=references%7CMainLayout::init) (1998) Contamination in Ontario farmstead domestic wells and its association with agriculture: 1. Results from drinking water wells. [*Jour. Contam. Hydro*.](http://www.refworks.com/refworks2/default.aspx?r=references%7CMainLayout::init) **32**

[Gosselin](http://www.refworks.com/refworks2/default.aspx?r=references%7CMainLayout::init) DC, [Headrick](http://www.refworks.com/refworks2/default.aspx?r=references%7CMainLayout::init) J, [Tremblay](http://www.refworks.com/refworks2/default.aspx?r=references%7CMainLayout::init) R,  [Chen](http://www.refworks.com/refworks2/default.aspx?r=references%7CMainLayout::init) XH, [Summerside](http://www.refworks.com/refworks2/default.aspx?r=references%7CMainLayout::init) S (1997) Domestic well water quality in rural Nebraska: Focus on nitrate-nitrogen, pesticides, and coliform bacteria. [*Ground Water Monitoring and Remediation*](http://www.refworks.com/refworks2/default.aspx?r=references%7CMainLayout::init). **17**(2)

Hancock CM, Rose JB, Callahan M (1998) *Cryptosporidium* and *Giardia* in US groundwater. *Jour. Am. Water Works Assoc*. **90**:58-61

[Hunt RJ](http://www.refworks.com/refworks2/default.aspx?r=references%7CMainLayout::init), [Borchardt MA](http://www.refworks.com/refworks2/default.aspx?r=references%7CMainLayout::init), [Richards KD](http://www.refworks.com/refworks2/default.aspx?r=references%7CMainLayout::init), [Spencer SK](http://www.refworks.com/refworks2/default.aspx?r=references%7CMainLayout::init) (2010) Assessment of sewer source contamination of drinking water wells using tracers and human enteric viruses. [*Environ. Sci. Technol*.](http://www.refworks.com/refworks2/default.aspx?r=references%7CMainLayout::init) **44**(20):7956-7963

Isaac-Renton J, Blatherwick J, Bowie WR, Fyfe M, Khan M, et al (1999) Epidemic and endemic seroprevalence of antibodies to *Cryptosporidium* and *Giardia* in residents of three communities with different drinking water supplies. *Am. Jour. Trop. Med. Hyg*. **60**(4), 578–583

[Johnson TB](http://www.refworks.com/refworks2/default.aspx?r=references%7CMainLayout::init), [McKay LD](http://www.refworks.com/refworks2/default.aspx?r=references%7CMainLayout::init), [Layton AC](http://www.refworks.com/refworks2/default.aspx?r=references%7CMainLayout::init), [Jones SW](http://www.refworks.com/refworks2/default.aspx?r=references%7CMainLayout::init), [Johnson GC](http://www.refworks.com/refworks2/default.aspx?r=references%7CMainLayout::init) et al (2011) Viruses and bacteria in karst and fractured rock aquifers in East Tennessee, USA. [*Ground Water*](http://www.refworks.com/refworks2/default.aspx?r=references%7CMainLayout::init)*.* **49**(1):98-110

[Johnson GC](http://www.refworks.com/refworks2/default.aspx?r=references%7CMainLayout::init) (2002) Water quality of springs in the Valley and Ridge physiographic province in the Upper Tennessee River Basin, 1997. [U.S. Geological Survey Water-Resources Investigations Report 02-4180](http://www.refworks.com/refworks2/default.aspx?r=references%7CMainLayout::init)

[Karon AE](http://www.refworks.com/refworks2/default.aspx?r=references%7CMainLayout::init), [Hanni KD](http://www.refworks.com/refworks2/default.aspx?r=references%7CMainLayout::init), [Mohle-Boetani JC](http://www.refworks.com/refworks2/default.aspx?r=references%7CMainLayout::init), [Beretti RA](http://www.refworks.com/refworks2/default.aspx?r=references%7CMainLayout::init), [Hill VR](http://www.refworks.com/refworks2/default.aspx?r=references%7CMainLayout::init) (2011) Giardiasis outbreak at a camp after installation of a slow-sand filtration water-treatment system. [*Epidemiol. Infect*.](http://www.refworks.com/refworks2/default.aspx?r=references%7CMainLayout::init) **139**(5):713-717

[Kozlica J](http://www.refworks.com/refworks2/default.aspx?r=references%7CMainLayout::init), [Claudet AL](http://www.refworks.com/refworks2/default.aspx?r=references%7CMainLayout::init), [Solomon D](http://www.refworks.com/refworks2/default.aspx?r=references%7CMainLayout::init), [Dunn JR](http://www.refworks.com/refworks2/default.aspx?r=references%7CMainLayout::init), [Carpenter LR](http://www.refworks.com/refworks2/default.aspx?r=references%7CMainLayout::init) (2010) Waterborne outbreak of *Salmonella* I 4,[5],12:i:-. [*Foodborne Pathog. Dis*.](http://www.refworks.com/refworks2/default.aspx?r=references%7CMainLayout::init) **7**(11):1431-1433

[Kozuskanich J](http://www.refworks.com/refworks2/default.aspx?r=references%7CMainLayout::init), [Novakowski KS](http://www.refworks.com/refworks2/default.aspx?r=references%7CMainLayout::init), [Anderson BC](http://www.refworks.com/refworks2/default.aspx?r=references%7CMainLayout::init) (2011) Fecal indicator bacteria variability in samples pumped from monitoring wells. [*Ground Water*](http://www.refworks.com/refworks2/default.aspx?r=references%7CMainLayout::init). **49**(1):43-52

[Lambertini E](http://www.refworks.com/refworks2/default.aspx?r=references%7CMainLayout::init), [Spencer SK](http://www.refworks.com/refworks2/default.aspx?r=references%7CMainLayout::init), Kieke BA, [Loge FJ](http://www.refworks.com/refworks2/default.aspx?r=references%7CMainLayout::init), [Borchardt MA](http://www.refworks.com/refworks2/default.aspx?r=references%7CMainLayout::init) (2011) Virus contamination from operation and maintenance events in small drinking water distribution systems. [*Jour. Water. Health.*](http://www.refworks.com/refworks2/default.aspx?r=references%7CMainLayout::init) **9**(4):799-812

[Lindsey BD, Rasberry JS, Zimmerman](http://www.refworks.com/refworks2/default.aspx?r=references%7CMainLayout::init) TM (2002) Microbiological Quality of Water From Noncommunity Supply Wells in Carbonate and Crystalline Aquifers of Pennsylvania[USGS Report](http://www.refworks.com/refworks2/default.aspx?r=references%7CMainLayout::init) 2002

Locas A, Barthe C, Barbeau B, Carriere A, Payment P (2007) Virus occurrence in municipal groundwater sources in Quebec, Canada. *Can. Jour. Microbiol*. **53**:688–694.

Locas A, Barthe C, Margolin AB, Payment P (2008) Groundwater microbiological quality in Canadian drinking water municipal wells. *Can. Jour. Microbiol*. **54**(6):472-478

[Maheux AF](http://www.refworks.com/refworks2/default.aspx?r=references%7CMainLayout::init), [Huppe V](http://www.refworks.com/refworks2/default.aspx?r=references%7CMainLayout::init), [Bissonnette L](http://www.refworks.com/refworks2/default.aspx?r=references%7CMainLayout::init), [Boissinot M](http://www.refworks.com/refworks2/default.aspx?r=references%7CMainLayout::init), [Rodrigue L](http://www.refworks.com/refworks2/default.aspx?r=references%7CMainLayout::init), et al (2012) Comparative analysis of classical and molecular microbiology methods for the detection of *Escherichia coli* and *Enterococcus* spp. in well water. [*Jour. Environ. Monit*.](http://www.refworks.com/refworks2/default.aspx?r=references%7CMainLayout::init) **14**(11):2983-2989

Mroz RC, [Pillai SD](http://www.refworks.com/refworks2/default.aspx?r=references%7CMainLayout::init) (1994) Bacterial populations in the groundwater on the US-Mexico border in El Paso County, Texas. [*South. Med. Jour*.](http://www.refworks.com/refworks2/default.aspx?r=references%7CMainLayout::init) **87**(12):1214-1217

[National Center for Environmental Health](http://www.refworks.com/refworks2/default.aspx?r=references%7CMainLayout::init) (NCEH) (1998) A Survey of the Quality of Water Drawn from Domestic Wells in Nine Midwest States. [CDC Report](http://www.refworks.com/refworks2/default.aspx?r=references%7CMainLayout::init) 1998

[Olsen SJ](http://www.refworks.com/refworks2/default.aspx?r=references%7CMainLayout::init), [Miller G](http://www.refworks.com/refworks2/default.aspx?r=references%7CMainLayout::init), [Breuer T](http://www.refworks.com/refworks2/default.aspx?r=references%7CMainLayout::init), [Kennedy M](http://www.refworks.com/refworks2/default.aspx?r=references%7CMainLayout::init), [Higgins C](http://www.refworks.com/refworks2/default.aspx?r=references%7CMainLayout::init), et al (2002) A waterborne outbreak of *Escherichia coli* O157:H7 infections and hemolytic uremic syndrome: implications for rural water systems. [*Emerg. Infect. Dis*.](http://www.refworks.com/refworks2/default.aspx?r=references%7CMainLayout::init) **8**(4):370-375

[O'Reilly CE](http://www.refworks.com/refworks2/default.aspx?r=references%7CMainLayout::init), [Bowen AB](http://www.refworks.com/refworks2/default.aspx?r=references%7CMainLayout::init), [Perez NE](http://www.refworks.com/refworks2/default.aspx?r=references%7CMainLayout::init), [Sarisky JP](http://www.refworks.com/refworks2/default.aspx?r=references%7CMainLayout::init), [Shepherd CA](http://www.refworks.com/refworks2/default.aspx?r=references%7CMainLayout::init), et al (2007) A waterborne outbreak of gastroenteritis with multiple etiologies among resort island visitors and residents: Ohio, 2004. [*Clin. Infect. Dis*.](http://www.refworks.com/refworks2/default.aspx?r=references%7CMainLayout::init) **44**(4):506-512

Parry SM, Salmon RL, Willshaw GA, Cheasty T (1998) Waterborne outbreak of Norwalk virus gastroenteritis at a southwest US resort: role of geological formations in contamination of well water. *Lancet*. **337**(8751):1200-1204

[Parshionikar SU](http://www.refworks.com/refworks2/default.aspx?r=references%7CMainLayout::init), [Willian-True S](http://www.refworks.com/refworks2/default.aspx?r=references%7CMainLayout::init), [Fout GS](http://www.refworks.com/refworks2/default.aspx?r=references%7CMainLayout::init), [Robbins DE](http://www.refworks.com/refworks2/default.aspx?r=references%7CMainLayout::init), [Seys SA](http://www.refworks.com/refworks2/default.aspx?r=references%7CMainLayout::init), et al (2003) Waterborne outbreak of gastroenteritis associated with a norovirus. [*Appl. Environ. Microbiol.*](http://www.refworks.com/refworks2/default.aspx?r=references%7CMainLayout::init) ***69****(9):5263-5268*

[St-Pierre K](http://www.refworks.com/refworks2/default.aspx?r=references%7CMainLayout::init), [Levesque S](http://www.refworks.com/refworks2/default.aspx?r=references%7CMainLayout::init), [Frost E](http://www.refworks.com/refworks2/default.aspx?r=references%7CMainLayout::init), [Carrier N](http://www.refworks.com/refworks2/default.aspx?r=references%7CMainLayout::init), [Arbeit RD](http://www.refworks.com/refworks2/default.aspx?r=references%7CMainLayout::init), [et](http://www.refworks.com/refworks2/default.aspx?r=references%7CMainLayout::init) al (2009) Thermotolerant coliforms are not a good surrogate for Campylobacter spp. in environmental water. [*Appl. Environ. Microbiol.*](http://www.refworks.com/refworks2/default.aspx?r=references%7CMainLayout::init) **75**(21):6736-6744

Strauss B, King W, Ley A, Hoey JR (2001) A prospective study of rural drinking water quality and acute gastrointestinal illness. BMC Public Health. **1**:8

[Swistock BR](http://www.refworks.com/refworks2/default.aspx?r=references%7CMainLayout::init), [Clemens S](http://www.refworks.com/refworks2/default.aspx?r=references%7CMainLayout::init), [Sharpe WE](http://www.refworks.com/refworks2/default.aspx?r=references%7CMainLayout::init), [Rummel S](http://www.refworks.com/refworks2/default.aspx?r=references%7CMainLayout::init) (2013) Water quality and management of private drinking water wells in Pennsylvania. [*Jour. Environ. Health*](http://www.refworks.com/refworks2/default.aspx?r=references%7CMainLayout::init). **75**(6):60-66

[Swistock BR](http://www.refworks.com/refworks2/default.aspx?r=references%7CMainLayout::init), [Sharpe WE](http://www.refworks.com/refworks2/default.aspx?r=references%7CMainLayout::init) (2005) The influence of well construction on bacterial contamination of private water wells in Pennsylvania. [*Jour. Environ. Health*](http://www.refworks.com/refworks2/default.aspx?r=references%7CMainLayout::init). **68**(2):22-36

[Zimmerman TM, Zimmerman ML, Lindsey BD](http://www.refworks.com/refworks2/default.aspx?r=references%7CMainLayout::init) (2001) Relation between selected well-construction characteristics and occurrence of bacteria in private household-supply wells, south-central and southeastern Pennsylvania. [(Report 01-4206). New Cumberland, PA: U.S. Geological Survey, Water-Resources Investigations.](http://www.refworks.com/refworks2/default.aspx?r=references%7CMainLayout::init)
